# Model-driven survival prediction after congenital heart surgery

**DOI:** 10.1093/icvts/ivad089

**Published:** 2023-06-05

**Authors:** Christoph Zürn, David Hübner, Victoria C Ziesenitz, René Höhn, Lena Schuler, Tim Schlange, Matthias Gorenflo, Fabian A Kari, Johannes Kroll, Tsvetomir Loukanov, Rolf Klemm, Brigitte Stiller

**Affiliations:** Department of Congenital Heart Defects and Paediatric Cardiology, University Heart Center Freiburg—Bad Krozingen, Medical Center—University of Freiburg, Faculty of Medicine, University of Freiburg, Germany; Machine learning for medical applications, Averbis GmbH, Freiburg, Germany; Department of Paediatric Cardiology and Congenital Heart Disease Center for Child and Adolescent Health, Medical Center—University of Heidelberg, Faculty of Medicine, University of Heidelberg, Germany; Department of Congenital Heart Defects and Paediatric Cardiology, University Heart Center Freiburg—Bad Krozingen, Medical Center—University of Freiburg, Faculty of Medicine, University of Freiburg, Germany; Department of Congenital Heart Defects and Paediatric Cardiology, University Heart Center Freiburg—Bad Krozingen, Medical Center—University of Freiburg, Faculty of Medicine, University of Freiburg, Germany; Faculty of Psychology, Ruhr University, Bochum, Germany; Department of Paediatric Cardiology and Congenital Heart Disease Center for Child and Adolescent Health, Medical Center—University of Heidelberg, Faculty of Medicine, University of Heidelberg, Germany; Department of Cardiovascular Surgery, University Heart Center Freiburg—Bad Krozingen, Medical Center—University of Freiburg, Faculty of Medicine, University of Freiburg, Germany; Department of Cardiovascular Surgery, University Heart Center Freiburg—Bad Krozingen, Medical Center—University of Freiburg, Faculty of Medicine, University of Freiburg, Germany; Department of Cardiothoracic Surgery, Medical Center—University of Heidelberg, Faculty of Medicine, University of Heidelberg, Germany; Department of Cardiovascular Surgery, University Heart Center Freiburg—Bad Krozingen, Medical Center—University of Freiburg, Faculty of Medicine, University of Freiburg, Germany; Department of Congenital Heart Defects and Paediatric Cardiology, University Heart Center Freiburg—Bad Krozingen, Medical Center—University of Freiburg, Faculty of Medicine, University of Freiburg, Germany

**Keywords:** Machine learning, Survival prediction, Congenital heart surgery, Paediatric intensive care

## Abstract

**OBJECTIVES:**

The objective of the study was to improve postoperative risk assessment in congenital heart surgery by developing a machine-learning model based on readily available peri- and postoperative parameters.

**METHODS:**

Our bicentric retrospective data analysis from January 2014 to December 2019 of established risk parameters for dismal outcome was used to train and test a model to predict postoperative survival within the first 30 days. The Freiburg training data consisted of 780 procedures; the Heidelberg test data comprised 985 procedures. STAT mortality score, age, aortic cross-clamp time and postoperative lactate values over 24 h were considered.

**RESULTS:**

Our model showed an area under the curve (AUC) of 94.86%, specificity of 89.48% and sensitivity of 85.00%, resulting in 3 false negatives and 99 false positives.

The STAT mortality score and the aortic cross-clamp time each showed a statistically highly significant impact on postoperative mortality. Interestingly, a child’s age was barely statistically significant. Postoperative lactate values indicated an increased mortality risk if they were either constantly at a high level or low during the first 8 h postoperatively with an increase afterwards.

When considering parameters available before, at the end of and 24 h after surgery, the predictive power of the complete model achieved the highest AUC. This, compared to the already high predictive power alone (AUC 88.9%) of the STAT mortality score, translates to an error reduction of 53.5%.

**CONCLUSIONS:**

Our model predicts postoperative survival after congenital heart surgery with great accuracy. Compared with preoperative risk assessments, our postoperative risk assessment reduces prediction error by half. Heightened awareness of high-risk patients should improve preventive measures and thus patient safety.

**Figure:**
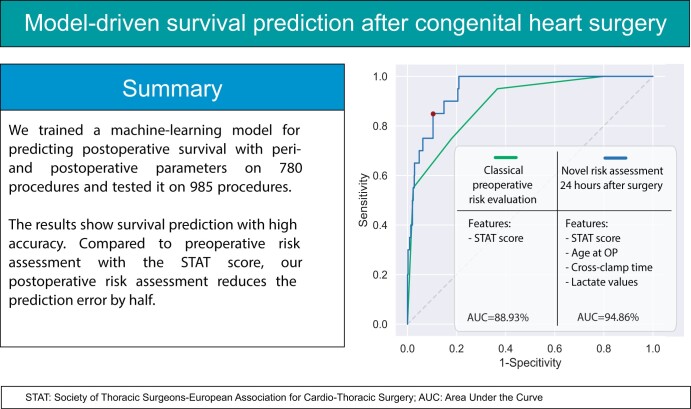

## INTRODUCTION

Assessing the risk of congenital heart surgery has evolved in recent years from being purely consensus-derived [1, 2] to being based on empirical tools such as the Society of Thoracic Surgeons–European Association for Cardio-Thoracic Surgery (STAT) congenital heart surgery mortality categories [3]. The STAT score is a well-established mortality risk estimation empirically founded on data from 77,294 operations. The score ranges from 1 to 5 for each procedure, where a higher score is associated with a higher mortality rate. In a validation sample of another 20,000 operations, O’Brien *et al.* clearly demonstrated that the STAT score is highly predictive of the actual outcome. They also showed that patient-level factors (i.e. age, weight and preoperative length of stay) clearly improved the predictive performance. The influence of age is in alignment with other studies that found that young age at surgery is associated with poor outcome, especially in neonates [4, 5]. A follow-up study analysed patient-specific preoperative factors in more detail and identified various risk factors such as mechanical circulatory support, renal dysfunction, shock and mechanical ventilation, among others, suggesting that more complex risk models should consider these factors [6].

All the existing approaches predict the mortality risk solely on the basis of preoperative information. In contrast, we propose also including the individual peri- and postoperative course of these children. Our goal is to enable a more accurate assessment of the probability of survival as early as 24 h after cardiac surgery to increase patient safety through primary and secondary prevention. Building on the STAT score’s well-established preoperative estimate of relative risk for in-hospital mortality after surgery, we have added 2 known and readily available operative risk markers to train a machine-learning model to further improve prediction results: First, the duration of the aortic cross-clamp time is well known to correlate with postoperative complication rates [7, 8]. Second, elevated lactate levels after the operation, especially if they persist during the postoperative period, are known to be a reliable predictor for a poor outcome [9, 10]. We hypothesized that a risk assessment that considers these 2 additional factors will reveal statistically significantly higher predictive power than a risk assessment solely based on preoperative factors.

In a joint effort, the departments of paediatric cardiology at Freiburg and Heidelberg University Hospitals trained and tested a model to predict postoperative survival within the first 30 days after the operation. We used data from Freiburg to develop and train the model and the data from Heidelberg to validate its predictions.

## PATIENTS AND METHODS

### Ethics statement

Approval for the study was obtained from the institutional research boards/ethics committees of Freiburg University (local IRB number 21–1676) and Heidelberg University (local IRB number S074/2022), along with a waiver of written consent. The study was registered at the German Clinical Trials Register with the identification number DRKS00028551 on 11 March 2022.

### Study design and population

Our study population consists of 780 and 985 patients from the departments of paediatric cardiology and cardiac surgery in Freiburg and Heidelberg, respectively, who underwent congenital heart surgery on cardiopulmonary bypass between January 2014 and December 2019. Patients who spent less than 24 h in the paediatric cardiac intensive care unit (PCICU) after surgery were excluded. Also, in children undergoing multiple operations, only the last stay in the PCICU was taken into account, because it rendered the question of previous survival obsolete. After the selection process, 495 participants from Freiburg remained as our training and 961 participants from Heidelberg as our test population. See Fig. S1 for the selection process of all participants. No patient died during or within 24 h after surgery. All children enrolled were between 1 day and 18 years of age.

### Machine-learning model development and evaluation

#### Parameters and features

This study uses 5 features based on 4 parameters that have all been studied as known risk factors for dismal outcome after an operation:

1. The STAT score, attributing a relative risk of in-hospital mortality based on the main congenital heart surgery procedure, ranging from 1 (0.8%) to 5 (23.1%) [3].
2. Age in days at the time of surgery.
3. Duration of aortic cross-clamp time in minutes.
4. Serum lactate levels in mmol/L. Based on this parameter, we computed 2 features, namely, the mean lactate values 8 and 24 h after the operation.

**Figure 1:**
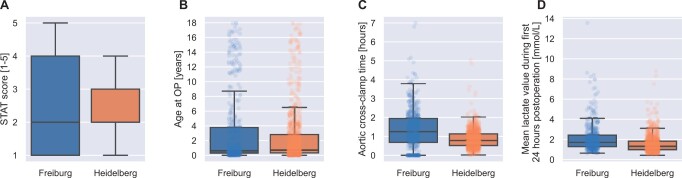
Overview of the features of both centres. Distributions are depicted as box-and-whisker plots. The box shows the interquartile range and the whiskers show the end of the box plus the 1.5⋅interquartile range but is limited to the lowest/highest data point. OP: operation; STAT: Society of Thoracic Surgeons–European Association for Cardio-Thoracic Surgery.

Whereas the STAT score and age are available before surgery, the aortic cross-clamp time is available right after the operation, and mean lactate values are available 8 h and 24 h after surgery, respectively. Thus, all features are available 24 h after the operation. Box-and-whisker plots for 4 different parameters (STAT score, age, aortic cross-clamp time, mean lactate values) and the data centres are illustrated in Fig. 1.

For each feature and both centres, the amount of missing data points was less than 5% of the total points. In case a value was missing, we imputed the median value of all patients at the clinic to which the sample belonged.

#### Logistic regression model

To identify and describe the relationship between the 5 features and the binary target variable *y* (postoperative survival after 30 days), we chose a binary logistic regression model. The output of the logistic regression model is a value in [0, 1]. To predict a specific class, one imposes a threshold and assigns outcomes above that threshold to the 1 class and outcomes below that threshold to the other class.

#### Class weighting scheme

Fortunately, postoperative survival is by far the most frequent outcome. However, that makes it that much harder to train a machine-learning model to predict postoperative mortality, because this outcome is under-represented in the data. Therefore, the algorithm strives to minimize the overall error rate by implying cost-sensitive learning [11], which means that each false-negative result gets a higher penalty when training the model. The weighting of the data was chosen to be approximately proportional to the class ratio of the training data. In the Freiburg dataset, there were 466 survivors for every 29 decedents, resulting in an approximate overweighting of factor 16 for the deceased patients.

#### Model training and evaluation

We trained and evaluated the model in a two-step procedure. In the first step, we only considered Freiburg’s data to select the best model and the hyperparameters. At this stage, we used a leave-one-out cross-validation that chunks the data into several parts, so that exactly 1 deceased patient and some non-deceased patients are in each part. The model is then trained on all but 1 part and evaluated on the remaining part. This procedure is repeated until the model is evaluated once on each patient and the score of each part is averaged. This approach enables the model to be trained on many patients while testing it on unseen patients to obtain a realistic performance estimation. We tried different features, model architectures and hyperparameters until settling for the best performing features and model: a logistic regression model without regularization and a classification threshold of 0.4. For the Freiburg dataset, we report on the leave-one-out cross-validation performance of that model.

In the second step, we took the optimized features and model architecture, trained it on the complete Freiburg dataset and evaluated it on the complete dataset of Heidelberg's department of paediatric cardiology to test its generalizability.

Regarding evaluation metrics, we report on specificity (“if the model predicts that a patient will die, how often is it correct?”) and sensitivity (“how many of the deceased patients are detected?”) and also display the confusion metrics. We also computed the area under the curve (AUC) of the receiver operating characteristic curve as a threshold-agnostic metric to capture how well the model’s probability output separates the 2 target classes. This value is between 0 and 1 (higher is better) with a change level of 0.5.

### Statistical evaluation

We used a randomized permutation test [12] to compute whether different feature sets led to a statistically significantly different classification performance. For that, we always made pairwise comparisons of a logistic regression model A based on a certain subset of features and another model B based on another subset of features. The null hypothesis is that both models perform equally well. To assess the null hypothesis, we computed the test statistics as the observed AUC difference between the 2 models. Then, we repeatedly applied a random permutation of the classifier output, where we assigned each output randomly to 1 of 2 groups. For each group, we then computed the AUC and the difference between both AUC values. By repeating this process 1000 times, we obtained a distribution over AUC differences. If the null hypothesis holds, then the observed difference should not statistically significantly differ from this distribution. Hence, we reject the null hypothesis if the observed value is outside the [2.5%, 97.5%] interval of the distribution. This corresponds to an alpha level of 0.05.

### Software implementation

A custom relational database based on FileMaker Pro 12 (Claris International Inc., Santa Clara, CA, USA), developed by the Department of Paediatric Cardiology Freiburg, was used to collect all intensive care unit data, aggregate clinical information and capture clinical information system parameters, such as aortic cross-clamp time and lactate values.

Python programming language (3.8.5) was used for the data processing, modelling, visualization and statistical assessment. More specifically, data preprocessing was realized using the library pandas (1.2.4) and traces (0.6.0). Statsmodels (0.13.1) and scikit-learn (0.24.2) were utilized for training and evaluating the logistic regression and random forest models. Visualizations were done with seaborn (0.11.1) and matplotlib (3.4.2). The bootstrapping test is based on a custom Python implementation.

## RESULTS

### Model performance

Our logistic regression model predicted postoperative survival after 30 days with high reliability. On the Freiburg dataset, the leave-one-out cross-validation showed an AUC of 91.45%, 80.90% specificity and 93.10% sensitivity. Notably, this high sensitivity translates to only 2 false negatives in the Freiburg dataset, i.e. deceased patients whom the model did not identify as such. The complete results for the Freiburg dataset are shown in Supplementary Fig. S2.

**Figure 2:**
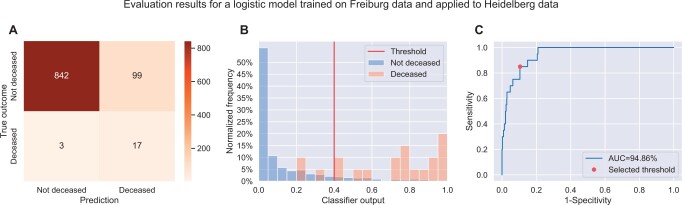
Evaluation results of the logistic regression model trained on the complete Freiburg dataset and tested on the Heidelberg dataset. (**A**) The confusion matrix showing the true positives (17), false positives (99), false negatives (3) and true negatives (842). (**B**) The classifier output values grouped by the actual outcome. The red vertical line indicates the chosen threshold of 0.4. Outputs above the threshold are mapped to “deceased” and others to “non-deceased”. (**C**) The receiver-operator curve shows the trade-off between sensitivity and specificity and the selected threshold. The area under the curve (AUC) accumulates to 94.86%.

Importantly, our model delivered excellent generalization results when applied to the Heidelberg dataset. The AUC surpassed that from Freiburg with 94.86%, while specificity was 89.48% and sensitivity 85.00%, slightly below that of Freiburg. As the confusion matrix depicts (Fig. 2A), this translates to a total of 3 false negatives and 99 false positives. The output probability clearly separates the 2 classes (Fig. 2B). All patients with a classifier output below 0.2 survived the operation, and most patients with a classifier output above 0.7 died. In between these 2 values, the model output does not clearly predict the outcome. The threshold of 0.4 leads to a reasonable trade-off between specificity and sensitivity (Fig. 2C). We assessed the model's calibration in Supplementary Fig. S3. It shows that for very high and very low probabilities, our model correctly identifies the outcomes. For probabilities in between, our model tends to overestimate the mortality risk.

### Odds ratios of the model coefficients

**Figure 3:**
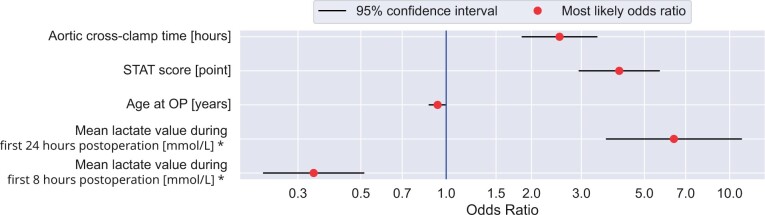
The influence of a change in model coefficients on the mortality odds ratio (OR). A 1-point change in the specified unit changes the OR by the depicted value (red dot). We depicted the 95% confidence interval of the OR estimation with a red horizontal line. Because both features regarding lactate values are closely correlated, these results need to be interpreted with caution. STAT: Society of Thoracic Surgeons–European Association for Cardio-Thoracic Surgery.

The influence of each model coefficient on postoperative mortality within 30 days after surgery was calculated as an odds ratio (Fig. 3). We observed the statistically highly significant impact of the STAT mortality score, whereby each point increased the mortality odds ratio (OR) by 4.08. Aortic cross-clamp time also proved to be a statistically highly significant risk factor, where each hour longer of aortic cross-clamp time increases the mortality OR by 2.51. Interestingly, the influence of patient age was statistically barely significant, where each year lowered the mortality risk by an OR of 0.93. Please note that the assumption of a constant OR for age is not given. With that, the feature “age” cannot be modelled adequately by the model and the OR of age has only limited validity.

Interpreting the OR of the lactate values is more difficult (see discussion) because both features, mean lactate after 8 and 24 h, are highly correlated (r_Pearson_ = 0.825).

The model coefficient and complete log odds ratios are found in Table S1.

### Influence of the features on the model performance

**Figure 4:**
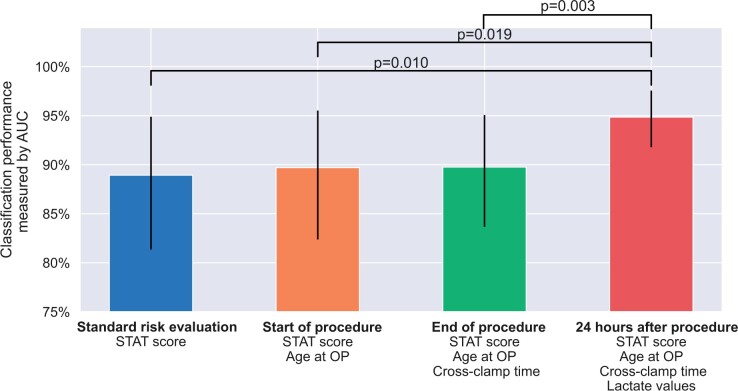
Evaluations for different logistic regression models trained at different time points during the operative timeline. From left to right, the models were trained with an increasing number of features, starting from only the STAT score (blue bar) to the complete feature set (red bar). The statistical significance of all pairwise AUC performance differences was calculated using a permutation test (see Methods). The 95% confidence interval of the AUC was estimated using bootstrapping in which the data points from the Heidelberg test were randomly resampled with replacement 1000 times. Only the statistically significant differences (*P* < 0.05) are displayed (raw *P*-values). All models used the same hyperparameters used in our final model and were trained on the complete Freiburg dataset and applied to the Heidelberg dataset. AUC: area under the curve; OP: operation; STAT: Society of Thoracic Surgeons–European Association for Cardio-Thoracic Surgery.

Based on the availability of parameters over time, we compared the performance of our risk prediction before, at the end of and 24 h after bypass surgery (see Fig. 4). Our results show that the STAT score alone already has high predictive power, with an AUC of 88.9%. Although the age at surgery and the aortic cross-clamp time add relatively little performance gain, the complete model including lactate values reveals a statistically significantly better performance with an AUC of 94.86%. This translates to a 53.5% error reduction when comparing the complete model to the preoperative model that uses only the STAT score. A permutation test was used to calculate pairwise AUC performance differences, showing that the postoperative mortality assessment including all parameters is statistically significantly superior to all other models with partial feature implementation.

## DISCUSSION

The logistic regression machine-learning model we propose predicts postoperative survival after 30 days for congenital heart surgery on cardiopulmonary bypass with high reliability. The promising results of the Freiburg training dataset, with an AUC of 91.45%, were surpassed by those of the Heidelberg test dataset, with an AUC of 94.86%, underscoring our model’s generalizability. Building upon and improving the STAT score’s risk assessment, the prediction error is approximately halved when perioperative model features are also considered.

Analysis of the model coefficients showed that the STAT score alone, as a well-established preoperative risk marker, had a statistically significant impact on survival prediction results, with a 4.08 increase in the mortality OR for each incremental point. Another preoperatively available parameter, namely age at time of surgery, proved to be less of a determinant than expected, resulting in a reduction in the mortality OR of only 0.93 for each year of age.

In combination with postoperative risk markers, our model demonstrates statistically significantly improved prediction accuracy, especially 24 h after surgery when mean serum lactate levels are considered. Each additional hour of aortic cross-clamp time resulted in an increase in the mortality OR of 2.51.

Because the mean serum lactate values at 8 and 24 h correlate so closely, their ORs (of 0.34 and 6.36) should be analysed jointly. There are 2 lactate profiles that lead to a high risk. If a patient maintains a constant, generally high lactate value, then the mean lactate values at 8 and 24 h would be the same, and the resulting OR would be the end product of the 2 individual ORs (joint OR: 0.34 * 6.36 = 2.16), meaning that a rise in the mean lactate value by 1 mmol/L over the first 24 h would increase the mortality risk by a factor of 2.16.

The second risk profile includes those patients whose lactate values during the first 8 h are low, but then rise afterwards. This would result in a high mean lactate value during the 24 h post operation. Our model would classify this latter profile as a risk profile because the OR for the 24 h mean lactate value is much higher than the OR for the first 8 h.

We chose this logistic regression model for various reasons, but the most important one was because it delivered the highest predictive performance in our experiments. We ran a comparison with an optimized random forest model, which is a more versatile and complex model, but its performance was worse (see Supplementary Chapter 1). In addition, logistic regression has the advantage that it predicts both a specific outcome and outputs a probability value that helps us to judge its confidence. Another advantage is that its model coefficients can be partially interpreted (also see limitations below) so that we are able to infer how each model coefficient is affecting the final prediction using the OR. Finally, the relatively simple, standard model architecture makes it easy to implement it in any clinical setting.

The current consensus-based risk evaluation methods for bypass surgery in children are static in their assessment, with no means of adjusting for patients' individual peri- and postoperative courses [1, 2]. A machine-learning model, such as the one presented here, successfully captures and incorporates perioperative information, thus predicting risk more accurately and, moreover, providing important insights for clinical decision making [8]. By emphasizing high-risk patients, known causes of increased postoperative risk such as delayed sternal closure, sepsis, chylothorax, diaphragmatic paresis, pulmonary hypertension, renal failure and arrhythmia [13–15] could be counteracted by timely secondary preventive measures or, wherever possible, avoided altogether by primary prevention.

Tabbutt *et al.* introduced the PC4 CICU Post-Surgical Mortality Model, which also shows high discrimination for survival prediction. With the aim of creating a benchmark for treatment in the PCICU, their risk assessment is based on 11 parameters obtained 2 h after arrival in the intensive care unit [15]. In comparison, our model relies on fewer elements tested 24 h after the operation, with a focus on practical bedside implementation.

Another major advantage is the reproducibility of making a reliable postoperative survival prediction irrespective of the physician's clinical experience and training level. The experience-independence of algorithm-assisted diagnosis has been well established via deep learning, from detecting polyps and adenomas during colonoscopy [16] to screening for melanomas [17]. This is not to say that machine learning in medical applications in general and in predicting postoperative survival in particular should replace clinical judgement and reasoning. However, we strongly believe that the resulting opportunities should be used as a useful bedside tool to enable better clinical care.

Future work should focus on testing the proposed model on a larger sample involving more cardiac centres, because the STAT score is based on over 77,000 operations. A larger number should allow the consideration of comorbidities to further improve survival estimates.

### Limitations of the model

The high data efficiency of the logistic regression model is based on strong assumptions that are partially violated for our dataset. Namely, the influence of 1 year of age difference on mortality is not constant—the decrease in risk between 0 and 1 years is much higher than the decrease from 17 to 18 years. In addition, the 2 lactate features are highly correlated, which hinders the interpretation of the model coefficients as ORs.

Examining our model’s incorrect predictions, analysis of the Heidelberg dataset yielded a total of 3 false-negative results, corresponding to a sensitivity of 85.00% at the chosen classification threshold. On closer examination of these type II errors, the children’s history revealed severe comorbidity or postoperative complications in each case.

The Heidelberg test data’s false-positive results accounted for 99 cases, with a specificity of 89.48% at the classification threshold. Reviewing these type I errors by inspecting 3 patients to whom the model attributed a poor outcome with the highest confidence, we detected a high-risk constellation in very young patients undergoing surgery, each with the highest STAT score of 5.

For more details on the clinical history of the aforementioned incorrect predictions, see Supplementary Chapter 2.

These examples of false predictions demonstrate that physicians' expertise and clinical judgement are and will remain crucial for the foreseeable future. Individual medical history is not considered in our model’s survival assessment. There is an obvious trade-off between a more accurate prognosis and the feasibility of collecting the required parameters in clinical practice.

## CONCLUSION

The machine-learning model we have created predicts postoperative survival after congenital heart surgery with great accuracy, statistically significantly improving the precision of predicted outcome in comparison to known risk mediators, while reducing prediction error by half in comparison to preoperative risk assessment.

## Supplementary Material

ivad089_Supplementary_Data

## Data Availability

In accordance with the approval of the ethics committees to protect the privacy of the participants, the data underlying this article cannot be made publicly available. The data will be shared with the corresponding author upon justified request.

## Abbreviations

AUC: Area under the curve
OR: Odds ratio
PCICU: Paediatric cardiac intensive care unit
STAT: Society of Thoracic Surgeons–European Association for Cardio-Thoracic Surgery
