# Supplementary material for: Model-driven survival prediction after congenital heart surgery
Source: Interdiscip Cardiovasc Thorac Surg. 2023 Jun 5;37(3):ivad089. doi: 10.1093/icvts/ivad089 (PMC10493173; doi:10.1093/icvts/ivad089)
Supplement: ivad089_Supplementary_Data [file ivad089_supplementary_data.zip › ICVTS_Supplementary_figures_1to3.docx]

# Supplementary Figures

## **Figure S1:**

##
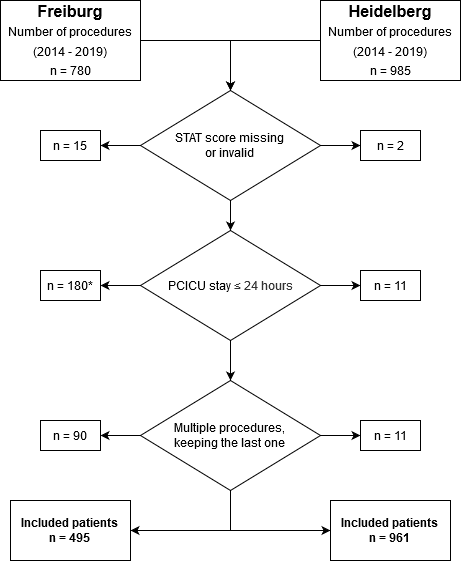


## Flowchart indicating the patient selection process. *Due to fast-track with early extubation, 180 children were transferred to the regular ward within 24 hours after bypass surgery.

## **Figure S2:**

## **
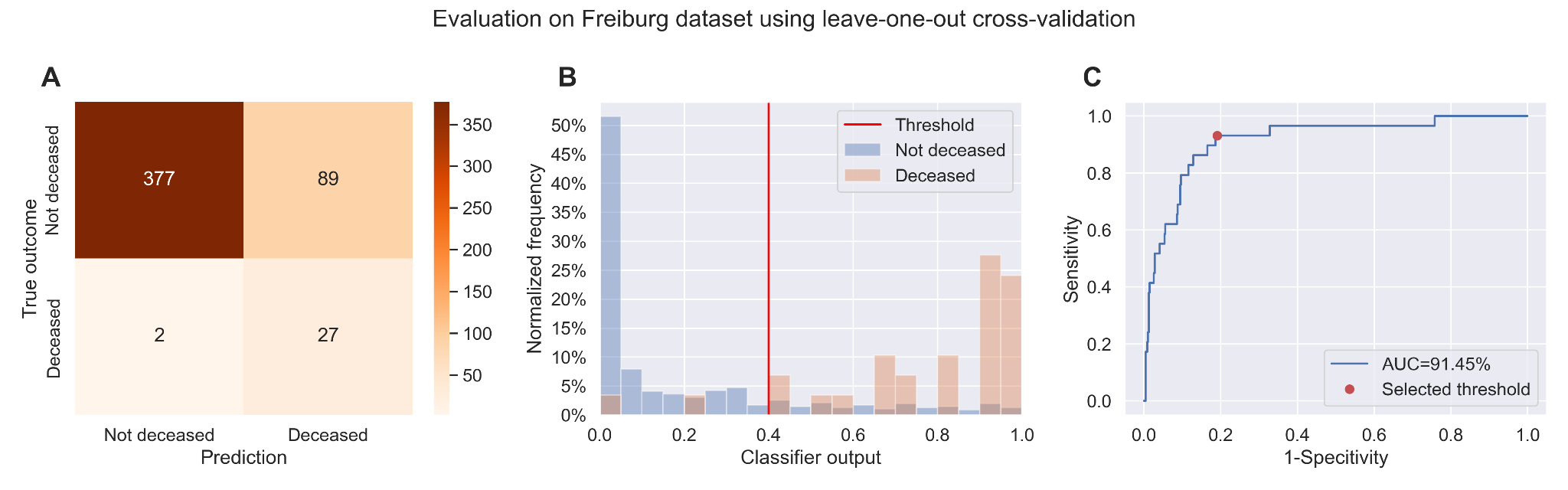
**

Performance evaluation on the Freiburg dataset using a leave-one-out cross-validation. For an explanation of the subplots, please see Figure 2.

## **Figure S3:**

**
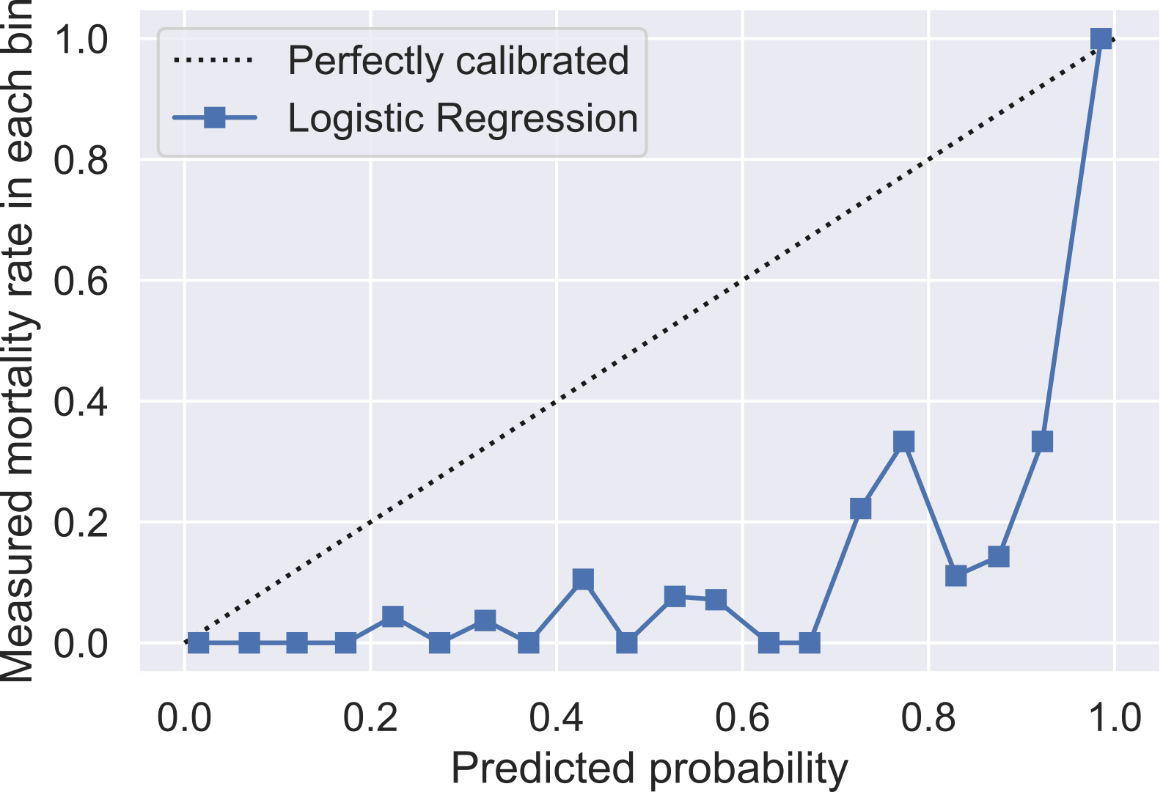
**

Calibration plot showing how the predicted mortality probability (confidence) on the x-axis corresponds to the measured mortality rate on the y-axis. An optimally calibrated model would follow the dashed line. For very high and very low probabilities, our model correctly identifies the outcomes. For probabilities in between, our model tends to overestimate the mortality risk.
