# Supplementary material for: Model-driven survival prediction after congenital heart surgery
Source: Interdiscip Cardiovasc Thorac Surg. 2023 Jun 5;37(3):ivad089. doi: 10.1093/icvts/ivad089 (PMC10493173; doi:10.1093/icvts/ivad089)
Supplement: ivad089_Supplementary_Data [file ivad089_supplementary_data.zip › ICVTS_Supplementary_material.pdf]

Supplementary Material

Chapter S1: Comparing the logistic regression model to a random forest model

In this section, we compare our logistic regression model with an optimized random forest model. Random forests are known to be a versatile and strong baseline model that can cope both with categorical and numeric data. A random forest is an ensemble estimator that fits several decision tree classifiers on various sub-samples of the dataset. The final classification decision is then obtained by a voting mechanism of the individual trees.

Random forest models have many hyperparameters that need to be tuned to the problem at hand. For that reason, we conducted a grid search over the most important parameters. In analogy to our optimization of the logistic regression model, we tested all possible parameter combinations (540) in a leave-one-out cross-validation and chose the best configuration according to the highest AUC score (Table S1). We then retrained the model with the chosen parameter set on the complete Freiburg dataset and applied it to the Heidelberg dataset. We utilized the random forest implementation from sklearn v0.24.2.

Compared to the logistic regression model, the results were slightly worse on the Freiburg dataset (-1.14%) and much worse (-5.13%) on the Heidelberg test dataset (Table S2).

See Table S3 for details about the final logistic regression model.

Chapter S2: Insights into the clinical background of false-predictions

False-negative predictions

The Heidelberg dataset showed three false-negative results, corresponding to a sensitivity of 85.00% at the chosen threshold of 40%:

1. Patient one was a neonate at 13 days of age with a dextro transposition of the great arteries (dTGA), ventricular septal defect (VSD), atrial septal defect (ASD), persistent ductus arteriosus (PDA) and a coronary anomaly with intramural left anterior descending artery who underwent an arterial switch procedure after rashkind septostomy. STAT mortality category was 3, aortic cross-clamp time 34 minutes, mean lactate after 8 hours 2.3 mmol/L and after 24 hours 2.4 mmol/L respectively. The model's predicted mortality was 23%. Postoperative occlusion of the left anterior descending artery necessitated installing extracorporeal membrane oxygenation (ECMO) on day 20 after surgery, resulting in the myocardium's inadequate recovery and the child's consecutive death.

2. Patient two was an infant at 208 days of age presenting an atresia of the pulmonary valve with intact VSD, right ventricle-dependent coronary circulation, non-restrictive ASD and PDA in palliation stage one after a modified Blalock-Taussig shunt operation. The child then received a bidirectional Glenn anastomosis. STAT mortality category was 4, aortic cross-clamp time 6 minutes, mean lactate after 8 hours 0.9 mmol/L and after 24 hours 1.1 mmol/L respectively. The model's predicted mortality was 23%.

Right ventricle-dependent coronary circulation followed cardiac ischemia, hypotonia and aggravating episodes of cyanosis ultimately leading to the child's death on day 13 after surgery.

3. Patient three was a neonate at 17 days of age with a dTGA, non-restrictive VSD, ASD, coronary artery anomaly with small left anterior descending artery who underwent an arterial switch procedure. STAT mortality category was 4, aortic cross-clamp time 44 minutes, mean lactate after 8 hours 3.3 mmol/L and after 24 hours 3.2 mmol/L respectively. The model’s predicted mortality was 32%.

   Junctional ectopic tachycardia developed postoperatively which led to cardiopulmonary reanimation and consecutive ECMO and left-ventricular vent implantation. Reconstruction of the coronary ostium and stent implantation into the left anterior descending artery followed on day 1 after surgery. Due to emerging severe hypoxic encephalopathy, ECMO weaning was impossible and the child died 13 days after the initial surgery.

Exemplary false-positive predictions with

The Heidelberg test data showed 99 false-positive results, corresponding to a specificity of 89.48% at the chosen threshold of 40%. The following three examples are the false-positives predicted with the highest confidence:

1. A neonate with a predicted mortality of 92% had hypoplastic left heart syndrome with mitral atresia, hypoplastic aortic arch and VSD who underwent Norwood procedure (STAT score 5) with a Sano shunt at the age of 3 days. Aortic cross-

clamp time was 54 minutes, mean lactate after 8 hours 6.2 mmol/L and after 24 hours 5.1 mmol/L respectively.

2. A neonate whose predicted mortality was 93% suffered hypoplastic left heart syndrome with mitral insufficiency, subvalvular aortic stenosis, aortic atresia, VSD and ostium secundum ASD, underwent the Norwood procedure (STAT score 5) with a Sano shunt, atrioseptectomy and mitral-valve closure at the age of 7 days. Aortic cross-clamp time was 94 minutes, mean lactate after 8 hours 4.9 mmol/L and after 24 hours 4.0 mmol/L respectively.

3. A neonate with a predicted mortality of 94% had a borderline left ventricle with hypoplastic aortic arch and restrictive PDA who underwent the Norwood procedure (STAT score 5) with Sano shunt at the age of 5 days. Aortic cross-clamp time was 74 minutes, mean lactate after 8 hours 4.1 mmol/L and after 24 hours 3.6 mmol/L respectively.
