# Supplementary material for: Model-driven survival prediction after congenital heart surgery
Source: Interdiscip Cardiovasc Thorac Surg. 2023 Jun 5;37(3):ivad089. doi: 10.1093/icvts/ivad089 (PMC10493173; doi:10.1093/icvts/ivad089)
Supplement: ivad089_Supplementary_Data [file ivad089_supplementary_data.zip › ICVTS_Supplementary_tables_1to3.docx]

# Supplementary Tables

**Supplementary Table S1:** Tuning of the hyperparameters for the random forest analysis.

| **Parameter** | **Meaning** | **Range** | **Optimal parameter** |
| --- | --- | --- | --- |
| **n_estimators** | Number of trees in random forest | [100, 500, 1000] | 100 |
| **max_features** | Number of features to consider at every split | [1, 2] | 1 |
| **max_depth** | Maximum number of levels in tree | [1, 2, 3, 4, 5] | 5 |
| **min_samples_split** | Minimum number of samples required at each leaf node | [2, 5, 10] | 2 |
| **min_samples_leaf** | Method of selecting samples for training each tree | [1, 2, 4] | 1 |
| **class_weight** | Method of weighting different classes | [None, "balanced"] | None |
| **threshold** | Similar to the logistic regression, this determines which fraction of trees needs to predict “deceased” in order for the model to predict “deceased” | manual selection | 0.05 |

**Supplementary Table S2:** Results of the random forest test and training data analysis.

| **Metric** | **Freiburg dataset** | **Heidelberg dataset** |
| --- | --- | --- |
| AUC | 90.31% | 89.73% |
| Specificity | 75.11% | 79.91% |
| Sensitivity | 82.76% | 80.00% |

**Supplementary Table S3:** Details about the final logistic regression model.

| **Feature** | **Unit** | **Coefficients** | **95% confidence interval** | **std err** | **z** | **P>\|z\|** |
| --- | --- | --- | --- | --- | --- | --- |
| Intercept |  | -7.993 | [-9.603, -6.384] | 0.821 | -9.735 | 2.14E-16 |
| Mean lactate until hour 8 | mmol/L | -1.077 | [-1.484, -0.669] | 0.208 | -5.180 | 2.22E-07 |
| Mean lactate until hour 24 | mmol/L | 1.850 | [1.302, 2.398] | 0.280 | 6.614 | 3.74E-11 |
| Age at OP | years | -0.070 | [-0.138, -0.001] | 0.035 | -1.987 | 4.70E-02 |
| STAT score | point | 1.406 | [1.081, 1.731] | 0.166 | 8.475 | 2.35E-17 |
| Aorta clamp time | hours | 0.921 | [0.618, 1.224] | 0.155 | 5.948 | 2.72E-09 |
