# Supplementary material for: Model-driven survival prediction after congenital heart surgery
Source: Interdiscip Cardiovasc Thorac Surg. 2023 Jun 5;37(3):ivad089. doi: 10.1093/icvts/ivad089 (PMC10493173; doi:10.1093/icvts/ivad089)
Supplement: ivad089_Supplementary_Data [file ivad089_supplementary_data.zip › ICVTS_Supplementary_tables_1to3.pdf]

Supplementary Tables

Supplementary Table S1: Tuning of the hyperparameters for the random forest analysis.

Parameter	Meaning	Range	Optimal parameter
**n_estimators**	Number of trees in random forest	[100, 500, 1000]	100
**max_features**	Number of features to consider at every split	[1, 2]	1
**max_depth**	Maximum number of levels in tree	[1, 2, 3, 4, 5]	5
**min_samples_split**	Minimum number of samples required at each leaf node	[2, 5, 10]	2
**min_samples_leaf**	Method of selecting samples for training each tree	[1, 2, 4]	1
**class_weight**	Method of weighting different classes	[None, "balanced"]	None
**threshold**	Similar to the logistic regression, this determines which fraction of trees needs to predict “deceased” in order for the model to predict “deceased”	manual selection	0.05

Supplementary Table S2: Results of the random forest test and training data analysis.

Metric	Freiburg dataset	Heidelberg dataset
AUC	90.31%	89.73%
Specificity	75.11%	79.91%
Sensitivity	82.76%	80.00%

Supplementary Table S3: Details about the final logistic regression model.

Feature	Unit	Coefficients	95% confidence interval	std err	z	P>|z|
Intercept		-7.993	[-9.603, -6.384]	0.821	-9.735	2.14E-16
Mean lactate until hour 8	mmol/L	-1.077	[-1.484, -0.669]	0.208	-5.180	2.22E-07
Mean lactate until hour 24	mmol/L	1.850	[1.302, 2.398]	0.280	6.614	3.74E-11
Age at OP	years	-0.070	[-0.138, -0.001]	0.035	-1.987	4.70E-02
STAT score	point	1.406	[1.081, 1.731]	0.166	8.475	2.35E-17
Aorta clamp time	hours	0.921	[0.618, 1.224]	0.155	5.948	2.72E-09
